# Italian intersociety consensus statement on antithrombotic prophylaxis in hip and knee replacement and in femoral neck fracture surgery

**DOI:** 10.1007/s10195-010-0125-8

**Published:** 2011-01-19

**Authors:** F. Randelli, F. Biggi, G. Della Rocca, P. Grossi, D. Imberti, R. Landolfi, G. Palareti, D. Prisco

**Affiliations:** 1 Hip Department, Orthopedics and Trauma II, IRCCS Policlinico San Donato, S. Donato Milanese, Milan, Italy; 2 Orthopaedic and Trauma Department, Belluno Hospital, Belluno, Italy; 3 Anesthesia and Intensive Care Medicine, University of Udine, Udine, Italy; 4 Regional Anesthesia and Pain Therapy Department, IRCCS Policlinico San Donato, S. Donato Milanese, Milan, Italy; 5 Internal Medicine Department, University Hospital of Ferrara, Ferrara, Italy; 6 Department of Internal Medicine, University Hospital A. Gemelli, Rome, Italy; 7 Unit of Angiology and Coagulation Disease, AOU Sant’Orsola-Malpighi, University of Bologna, Bologna, Italy; 8 Department of Internal Medicine, University of Florence, Florence, Italy; 9 Thrombosis Regional Reference Centre, Careggi University Hospital, Florence, Italy

**Keywords:** Prevention of venous thromboembolism, Total knee replacement, Total hip replacement, Anticoagulant prophylaxis, Rivaroxaban, Dabigatran, Enoxaparin, Femoral neck fractures

## Abstract

Anticoagulant prophylaxis for preventing venous thromboembolism (VTE) is a worldwide established procedure in hip and knee replacement surgery, as well as in the treatment of femoral neck fractures (FNF). Different guidelines are available in the literature, with quite different recommendations. None of them is a multidisciplinary effort as the one presented. The Italian Society for Studies on Haemostasis and Thrombosis (SISET), the Italian Society of Orthopaedics and Traumatology (SIOT), the association of Orthopaedists and Traumatologists of Italian Hospitals (OTODI), together with the Italian Society of Anesthesia, Analgesia, Resuscitation, and Intensive Care (SIAARTI) have set down easy and quick suggestions for VTE prophylaxis in hip and knee surgery as well as in FNF treatment. This inter-society consensus statement aims at simplifying the grading system reported in the literature, and its goal is to benefit its clinical application. Special focus is given to fragile patients, those with high bleeding risk, and those receiving chronic antiplatelet (APT) and vitamin K antagonists treatment. A special chapter is dedicated to regional anaesthesia and VTE prophylaxis.

## Introduction

Venous thromboembolism (VTE) represents a problem of relevant clinical and social impact. Recent data indicate an incidence of VTE of approximately 900,000 cases per year in the USA and of approximately 770,000 in Europe; in addition, pulmonary embolism (PE) is the direct cause of almost 10% of in-hospital deaths [1].

Anticoagulant prophylaxis for preventing VTE is a well-established procedure in hip (HR) and knee (KR) replacement surgery and in treating femoral neck fractures (FNF). Several meta-analyses indicate that in patients undergoing this kind of prophylactic treatment, an important reduction in symptomatic VTE is seen, with no relevant increase in major bleeding events [1–5]. These observations led the American College of Chest Physicians to generate universally recognized grade Ia recommendations on the need to initiate prophylaxis before all HR surgical interventions and to prolong treatment during the following 5 weeks. At present, pharmacological and/or mechanical prophylaxis is started in all cases of major orthopedic surgery (MOS), including elective HR and KR, and FNF surgery, as well as in several other cases of fracture (high-impact trauma, multiple fractures, multiple trauma) [1].

The Italian Society for Studies on Haemostasis and Thrombosis (SISET) has been focussing its research efforts on this topic for many years [2, 3]. When the need to formulate practical recommendations arose in the world of orthopedics and traumatology, the Italian Society of Orthopaedics and Traumatology (SIOT) and the association of Orthopaedists and Traumatologists of Italian Hospitals (OTODI) identified SISET and the Italian Society of Anesthesia, Analgesia, Resuscitation and Intensive Care (SIAARTI) as their natural counterparts. This intersociety consensus statement aims at simplifying the grading system reported in the literature, and its goal is to improve its clinical application. For this reason, we believed that there was no need to define the strength of recommendations provided, as national and international dedicated guidelines already exist [1–5]. This statement is therefore addressed to the Italian scientific community and institutions with the aim of attaining good clinical practice in the profession.

The present statement will be published in the Journals of the different Societies participating in this consensus.

## Purpose

Four purposes have been identified:

1. Keeping patients as safe as possible concerning the possibility of a thromboembolic event as a potential sequela in case of HR, KR, or FNF surgery in adults.
2. Reducing the possible complications linked to antithrombotic prophylaxis following HR, KR, or FNF surgery as much as possible.
3. Providing all specialists involved with unequivocal indications on the types of antithrombotic prophylaxis to be followed, in keeping with data reported in the national and international literature and with the laws in force in Italy.
4. Supplying useful suggestions on daily clinical practice in all situations in which no clear evidence is provided.

## Patients

Patients were subdivided into three groups:

- Patients at high risk of VTE;
- Patients at high risk of bleeding;
- Particular or “fragile” patients requiring individualized treatment.

### Patients at high risk of VTE

All patients undergoing HR, KR, or FNF surgery are at high risk of VTE and must follow an antithrombotic prophylaxis protocol. In-depth hematological and instrumental screening in quest of additional risk factors for thromboembolism is not believed to be essential, as knowledge of these factors would not alter the prophylactic strategies. An exception is made for patients with past episodes of deep vein thrombosis (DVT) and/or pulmonary embolism, who require an individualized preventive/curative approach.

### Patients at high risk of bleeding

Patients at high risk of bleeding are described in Table 1.

**Table 1:** Patients at high risk of bleeding and patients who need careful evaluation for possible risk of bleeding

Patients at high risk of bleeding	Patients to be carefully evaluated for possible risk of bleeding
Prolonged PT (INR > 1.5)	Prolonged APTT (except antiphospholipid antibody syndrome)
Thrombocytopenia < 50,000/µl	
Known bleeding diathesis	Severe CRF (creatinine clearance < 30 ml/min)
Chronic liver disease with prior bleeding episodes	Family or personal history of major bleeding
Multiple trauma (ISS ≥ 15)	Concomitant use of drugs affecting hemostasis (e.g., antiplatelet drugs, anti-inflammatory drugs)

*PT* prothrombin time,*INR* International normalized ratio,*ISS* injury severity score,*APTT* antiplatelet treatment,*CRF* chronic renal failure

### Fragile patients

Fragile patients requiring individualized treatment are those who present with:

- Body weight <50 kg
- Age >75 years
- Moderate chronic renal failure (CRF) (creatinine clearance 30–50 ml/min)

The creation of a personalized, shared folder for thrombotic and hemorrhagic risk assessment and initiation of adequate thromboprophylaxis is suggested in all hospital settings. Furthermore, we recommend that the creation of this document be suggested by all administrations involved (hospital directorate, local health authority, regional administration, etc.).

## Type of prophylaxis

**Table:** 

Pharmacological	LMWH, FON, NOA, VKA, UH
Mechanical	Active (IPC, VFP) Passive (GCS)
Combined	Pharmacological + mechanical

*LMWH* low-molecular-weight heparin,*FON* fondaparinux,*NOA* new oral anticoagulants,*UH* unfractionated heparin,*VKA* vitamin K antagonists, *VFP* venous foot pump,*IPC* intermittent pneumatic compression,*GCS* graduated compression stockings

### Pharmacological prophylaxis

- Pharmacological prophylaxis is based on low-molecular-weight heparin (LMWH), fondaparinux (FON), and new oral anticoagulants (NOA).
- Aspirin must not be used for VTE prophylaxis, as indicated by its label and by current guidelines.
- Unfractionated heparin (UH) must not be used considering that its efficacy is lower than that of LMWH, it has a short half-life, and it more frequently induces thrombocytopenia.
- Vitamin K antagonists (VKA) should not be administered because they are difficult to manage and maintain within a range of therapeutic anticoagulation [International normalized ratio (INR) ranging between 2 and 3].

Exceptions are possible but must be evaluated on an individualized basis with the consultant cardiologist or an expert in hemostasis and thrombosis.

#### Low-molecular-weight heparin (LMWH)

Concerning HR and KR, no differences in efficacy and safety have been reported between LMWH preoperative and postoperative first administration (Table 2) [6, 7]. LMWH labels in Italy, however, require a preoperative first administration except for bemiparin and dalteparin (for the latter only in hip surgery).

**Table 2:** Dosage and time of administration of low-molecular-weight heparin (LMHW) available in Italy

Active principle	Brand name	Dosage and time of administration
Enoxaparin	Clexane^®^	4,000 IU 12 h before surgery, then 4,000 IU/day
Nadroparin	Fraxiparine^®^ Seleparin^®^	38 IU/kg 12 h before surgery and 12 h after, 38 IU/kg every 24 h during the 3 days following surgery, thereafter increasing the dose to 57 IU/kg/day
Dalteparin	Fragmin^®^	5,000 IU 8–12 h before surgery, then 5,000 IU/day. Alternatively 2 h, 500 IU 1–2 before surgery^a^ and 2,500 IU 8–12 h after, thereafter either 5,000 IU/day or (only in hip surgery) 2,500 IU 4–8 h after surgery then 5,000 IU/day
Bemiparin	Ivor^®^	3,500 IU 6 h after surgery, then 3,500 IU/day. Alternatively 3,500 IU 2 h before surgery^a^, then 3,500 IU/day
Parnaparin	Fluxum^®^	0.4 ml (4,250 anti-Xa IU) 12 h before surgery, then 0.4 ml (4,250 anti-Xa IU)/day
Reviparin	Clivarin^®^	0.4 ml (4,200 anti-Xa IU) 12 h before surgery, then 0.4 ml (4,200 anti-Xa IU)/day

^a^Although reported by the product label, this type of prophylaxis is not recommended

#### Fondaparinux (FON)

Fondaparinux has proved to be effective and safe in VTE prevention in HR, KR, and FNF (Table 3) [8]. In particular, FON has been reported to be more effective than LMWH (only demonstrated by decrease in phlebography-proven asymptomatic DVT) with modest, although statistically significant, increase in bleeding and need for transfusions (with no related increase in fatal hemorrhage, in critical organs, or need for reintervention).

**Table 3:** Dosage and time of administration of Fondaparinux

Active principle	Brand name	Dosage and time of administration
Fondaparinux	ARIXTRA^®^	2.5 mg at least 6 h after surgery, then 2.5 mg/day^a^ If creatinine clearance 20–50 ml/min 1.5 mg^b^

^a^In agreement with the latest edition of the American College of Chest Physicians (ACCP) guidelines [1], initiation may be postponed up to 24 h after the end of the intervention [9], although this has not been included in the label as yet

^b^According to the recent guidelines of the European Society of Anaesthesiology [10], FON is contraindicated if creatinine clearance < 30 ml/min

#### New oral anticoagulants (NOA)

New oral anticoagulants (dabigatran and rivaroxaban) have proved to be effective and safe in VTE prevention in HR and KR (Table 4) [11–16]. On the other hand, no direct comparison has ever been made between the two drugs, allowing for a definite confirmation of any different efficacy and safety. There is no evidence in the literature concerning the use of NOA in patients undergoing FNF surgery and concerning prolonged prophylaxis after KR; furthermore, experience in fragile patients is limited. Although these drugs do not require laboratory monitoring, they have been shown to prolong PT and APTT.

**Table 4:** Dosage and time of administration of available new oral anticoagulants (NOA)

Active principle	Brand name	Dosage and time of administration
Dabigatran^a^ (antifactor IIa)	Pradaxa^®^	110 mg 1–4 h after surgery, then 220 mg/day If age > 75 years or creatinine clearance 30–50 ml/min or amiodarone intake, 75 mg 1–4 h after surgery, then 150 mg/day
Rivaroxaban^b^ (antifactor Xa)	Xarelto^®^	10 mg 6–10 h after surgery, then 10 mg/day

^a^Dabigatran has proved not to be inferior to low-molecular-weight heparin (LMWH) both in terms of efficacy and safety. As concerns dabigatran, in the literature, there is no information available on patients undergoing regional anaesthesia [11, 12]

^b^Rivaroxaban has shown to have greater efficacy than LMWH, with overlapping safety [13–16]. An analysis performed after publication of rivaroxaban registration study confirmed its safety in patients undergoing neuraxial anesthesia

### Mechanical prophylaxis

Mechanical prophylaxis is based on the use of graduated compression stockings (GCS) and on intermittent pneumatic compression (IPC) devices [17]. GCS (thigh-to-foot or knee-to-foot) increase the effectiveness of pharmacological prophylaxis, must be used until recovery of good mobility with autonomous de-ambulation (best if used on both legs), must be correctly positioned avoiding the “tourniquet effect,” and must not be used in the presence of peripheral arterial disease or diabetic neuropathy. IPC devices (either sural or plantar) have a high efficacy and enhance the action of anticoagulant drugs, but there is a low compliance by nurses and patients as concerns their management.

## When should prophylaxis be started?

### Patients with femoral neck fracture (FNF)

Selection and initiation of the prophylactic treatment to be followed strongly depend on the adopted schedule:

- If surgery is performed on an emergency basis (within 24 h), LMWH may be used (starting 12 h before or 12 h after) or, alternatively, FON (starting at least 6 h after the end of the intervention and, in any case, within 24 h).
- If surgery is postponed, LMWH must be administered early. In this case, there is no information available on the possibility of initiating FON 6–8 h after the end of the intervention, thus producing a shift between the two anticoagulant drugs. At present, no recommendation can be made on this subject.
- NOA must not be used, as no study pertaining to FNF has been published.

### Patients candidate for hip (HR) and knee (KR) replacement

In the literature, no significant difference in efficacy and safety has been reported between preoperative and postoperative initiation of LMWH in HR and KR [1, 6, 7]. Consequently, the choice must be based on evidence reported in published studies as well as on what is indicated on LMWH labels, which in Italy require initiation of prophylaxis 12 h before surgery, except for dalteparin and bemiparin (Table 2). Both FON and NOA must always be started postoperatively (Tables 3, 4).

## How long should pharmacological prophylaxis last?

Concerning the duration of pharmacological prophylactic treatment, if LMWH is used, therapy should last a minimum of 10 days in all patients, with a strong recommendation to protract prophylaxis for 35 days after HR and FNF surgery and the suggestion – with a lower level of evidence – to protract treatment similarly in patients undergoing KR surgery [1, 18]. Regardless, in Italy, it is standard procedure to protract prophylaxis for 35 days even after KR surgery. This approach is also suggested for FON therapy. As far as NOA are concerned, indications on duration of treatment derive from registration studies and are reported on the labels of dabigatran and rivaroxaban:

- With dabigatran, duration is 4–5 weeks in HR and 10 days in KR surgery;
- With rivaroxaban, duration is 5 weeks in HR and 2 weeks in KR surgery.

The safety of 5-week treatment with NOA has been proven in HR studies, which suggest the reliability and feasibility of this prophylaxis duration after KR as well. Lastly, it must be remembered that further protraction of prophylaxis (longer than suggested duration) has to be addressed in patients who, for different reasons (prolonged recumbence, additional risk factors), are at risk of developing VTE complications for a longer period than usual.

## Anesthesia techniques and initiation of pharmacological prophylaxis

No particular problem is identified in relation to general anesthesia (GA). On the other hand, regarding regional anesthesia (RA), timing must be carefully respected with epidural or intrathecal anesthesia, whereas there are no contraindications in perineural block [19, 20]. It is widely accepted that RA reduces the risk of VTE and that the correct timing (prophylaxis/RA administration and, if present, catheter removal) is crucial to prevent complications. Actually, all anticoagulants used in VTE prevention in HR, KR, and FNF are closely related to the risk of developing epidural hematoma. In particular, upon removal of the epidural catheter, drug effectiveness, half-life (T_1/2_), and time to maximum concentration (T_max_) must be assessed: as a general rule, the recommendation is made to wait at least 2 half-lives before removal, resuming pharmacological prophylaxis after 8 h (period required for clot formation) minus T_max_.

To simplify:

- *LMWH and RA* [19, 20]
- T_1/2_: 4 h
- T_max_: 4 h

Last administration before catheter removal: at least 12 h.

First administration after catheter removal: at least after 6–8 h.

If LMWH is administered twice daily, either at the prophylactic or therapeutic dosage, 24 h must pass after catheter removal before proceeding with the following dose.

If traumatic puncture, consider the possibility of initiating prophylaxis after 24 h.

- *FON and RA* [21]
- T_1/2_: 17 h
- T_max_: 1 h

If FON is administered at the therapeutic dosage, no central block must be performed.

Last administration before catheter removal: at least 36 h.

First administration after catheter removal: at least after 12 h.

If traumatic puncture, consider the possibility of initiating prophylaxis after 24 h.

- *NOA and RA* [10]

As concerns the relationship between NOA and RA, there is no information available (randomized clinical studies) concerning timing and method of use; therefore, refer to what is reported on the product label:

- Dabigatran—not recommended in patients who must undergo anesthesia requiring the use of postoperative permanent epidural catheters, as no information is reported in the literature.
- Rivaroxaban—last administration 18 h before removal, resume administration 6 h after removal; recent guidelines of the European Society of Anaesthesiology suggest a longer period between last rivaroxaban dose and epidural catheter removal (22–26 h) [10].

## Anesthesia/patient correlation in antiplatelet treatment

See Table 5.

**Table 5:** Correlation between anesthesia and antiplatelet treatment (APT)

Regional anesthesia^a^		General anesthesia
Patients on APT with		Patients on APT
Acetylsalicylic acid (ASA): do not interrupt in case of secondary prevention (75–100 mg/day)	Ticlopidine—interrupt 10 days pre-op	GA always feasible
IIb/IIIa inhibitors Abciximab—RA contraindicated Eptifibatide—interrupt 8 h pre-op Tirofiban—interrupt 4 h pre-op	Clopidogrel—interrupt 7 days pre-op	Risk of surgical bleeding must always be considered before surgery

^a^APT, if no bleeding occurs, must be resumed the day following the intervention and, in the presence of epidural catheterization, after catheter removal

## Management of vitamin K antagonist (VKA) patients

The main purpose is leading patients to surgical intervention with an adequate hemostasis and reducing the risk of thromboembolism as much as possible.

### Femoral neck fracture (FNF) patients

Intervention should be delayed and INR measured: If INR > 2, administer vitamin K 10 mg in 100 ml of saline or glucose solution i.v. and measure INR every 6/8 h until INR < 2 is attained. If INR < 2, start LMWH administration at the prophylactic dose and timing (4,000–5,000 IU/day), plan surgical intervention as soon as possible, and request consultation by a cardiologist and/or by an expert in hemostasis and thrombosis to plan VKA resumption after surgery.

### Patients candidate for elective hip (HR) and knee (KR) replacement

Each hospital should have a written and shared protocol concerning the management of VKA patients who have to undergo major lower-limb orthopedic surgery; consultation by a cardiologist and/or an expert in hemostasis and thrombosis should be requested to prepare a personalized schedule addressing VKA interruption and resumption; the timing of surgery must be respected, and the procedure should not be delayed.

## Management of antiplatelet treatment (APT) patients

Aspirin administered as primary prevention must be interrupted 7 days before elective surgery, whereas it must be interrupted upon hospital admission in patients with FNF planned for surgery. Aspirin administration as secondary prevention (in patients with prior cardiovascular events) must be continued at the dose of 75–100 mg/day.

### Femoral neck fracture (FNF) patients

For FNF, APT patients should undergo surgery as soon as possible. For patients on clopidogrel or ticlopidine (or dual anti-aggregation), request consultation by a cardiologist and/or an expert in hemostasis and thrombosis.

### Patients candidate for hip (HR) and knee (KR) replacement

Administration of clopidogrel or ticlopidine must be interrupted 7 and 10 days before surgery, respectively, whereas in patients receiving dual anti-aggregation (aspirin and clopidogrel), surgery must be delayed if clopidogrel interruption is expected during the following months; if interruption is not expected, request consultation by a cardiologist and/or an expert in hemostasis and thrombosis. In all such patients, as a general rule, resume APT as soon as possible and regardless, once hemostasis is achieved.

## Patients at high risk of bleeding

In patients at high risk of bleeding, the absolute contraindication to pharmacological prophylaxis is represented by ongoing major bleeding. In this case, mechanical prophylaxis is indicated. The relative contraindication is applied to all the other conditions for which patients are at high risk of bleeding reported in Table 1. In these cases, pharmacological or transfusional correction of the hemostatic defect is recommended whenever indicated and feasible, considering mechanical and/or dedicated pharmacological prophylaxis (dose reduction, postoperative initiation).

## Special cases or fragile patients

Providing precise directives for managing such patients goes beyond the purpose of this consensus statement; the only indication is to pay extreme attention to them and to request consultation by an expert on hemostasis and thrombosis. Management of anticoagulant drugs in obese patients [body mass index (BMI) > 30] is not considered to be different from what occurs with other patients. In patients with renal failure, labels of the single drugs administered must be referred to, and careful clinical monitoring must follow.

## General considerations

- Postoperative mobilization must be started as soon as possible.
- Bed-rest patients should receive lower-limb mobilization exercises.
- General practitioners and patients should be informed how to recognize signs and symptoms of DVT and PE, how to correctly manage home prophylaxis, and about the risks of omitting it.
- Pharmaceutical companies and regulatory authorities (Italian Medicines Agency, etc.) should keep labels updated in agreement with scientific evidence reported in the literature.

## Conclusions

This document represents a consensus statement of Italian experts, with information based on scientific knowledge and labels available during the summer of 2010, and it will be disseminated by the four societies via different modalities (society journals, society Web sites, symposia organized within national congresses, etc.). A periodical revision of this document is expected, which will be of particular importance for the use of new anticoagulant drugs currently undergoing clinical development, some of which (edoxaban, betrixaban, and others) are still undergoing a preliminary trial phase. For other drugs (apixaban), studies are already available [22, 23] that prove their efficacy and safety in VTE prevention in HR and KR surgery. It is therefore likely that the number of drugs available for this type of prophylaxis will increase in the near future.
